# Response Surface Approach to Optimize the Conditions of Foam Mat Drying of Plum in relation to the Physical-Chemical and Antioxidant Properties of Plum Powder

**DOI:** 10.1155/2021/3681807

**Published:** 2021-12-20

**Authors:** Saad A. D. Sifat, Anuva T. Trisha, Nurul Huda, Wahidu Zzaman, Norliza Julmohammad

**Affiliations:** ^1^Department of Food Engineering and Tea Technology, Shahjalal University of Science and Technology, Sylhet 3114, Bangladesh; ^2^Faculty of Food Science and Nutrition, Universiti Malaysia Sabah, Jalan UMS, 88400 Kota Kinabalu, Sabah, Malaysia

## Abstract

This research was done to optimize the influence of various egg albumin (EA) concentrations of 2, 4, and 6% as a foaming agent and whipping times of 5, 10, and 15 minutes, on physicochemical and antioxidant properties of plum powder produced using response surface methodology (RSM). Physical properties of the foam such as density, porosity, and expansion were determined. After drying and powder manufacturing, physical properties, namely, the water absorption index (WAI) and water solubility index (WSI), as well as chemical characteristics such as pH, titratable acidity, and browning index, were assessed. Finally, antioxidant capabilities such as the total phenol content (TPC), DPPH scavenging activity, beta carotene, and total flavonoid content (TFC) were measured. According to the findings, both whipping duration and EA concentration had a substantial effect on the foam forming characteristics. Foam expansion increased significantly with EA concentration and whipping time increase, but foam density exhibited an inverse relationship as expected. Increases in EA concentration and whipping duration both raised pH values whereas titratable acidity exhibited an inverse tendency as variable quantity rose. The browning index dropped as EA concentration increased. Antioxidant qualities were retained in dried sample powder as compared with the fresh sample, and they were also altered by variable changes. Overall, a 4% EA concentration for 10 to 15 minutes produced the best dehydration effects with the most antioxidant retention.

## 1. Introduction

Plum fruit is full of micronutrients and antioxidants which can be of numerous health benefits to the consumers. Three plums fruits provide 30% vitamin C of our daily requirement. This colorful and succulent fruit belongs to the subgenus Prunus under the Rosaceae family. Plums come in a wide variety of colors and sizes with the taste ranging from sweet to tart with a super juicy mouthfeel when consumed fresh. Plums can be consumed farm fresh, dried which are also known as prunes, and made as jam or jelly with other delicacies, mainly commercialized for prunes, fruit juices, and sweet syrup; plums are also fermented into plum wines. With China being in the lead position of its global domestic production, plum is produced largely in Romania, Serbia, United States, Poland, Iran, Turkey, and all EU countries producing about 15% of the world's production [1]. As a nutrient-rich functional food well known for its disease-preventing capabilities [2], plums are exported all over the world form the producer countries.

Due to the high perishable nature of plums with 87% water content, plums need to be dried and preserved, especially for exportation safety and storage and to reduce transportation loss [3]. As plums are seasonal fruits, developing new and attractive powdery elements and additives is necessary to enjoy them throughout the year. By consideration of weight, prunes are higher in calories, fiber, and carbohydrates than fresh plums. Prunes and products obtained from them have been thoroughly examined for their numerous health benefits. Dried plums are well known for their ability to remedy constipation due to their high dietary fiber and pectin contents. Including laxative properties, prunes are also attributed to having the ability to reduce bone loss [4] and age prevention properties [5] and decrease osteoporosis and also blood pressure [6]. Prunes are an abundant source of antioxidants, especially polyphenols and flavanols. The major groups of polyphenols consist of five polyphenolic acids, among which 3-feruloylquinic acid is the most prominent one. Anthocyanins make up 2% of the total polyphenols found in prunes [7]. Prunes also have very high radical scavenging activity, higher than any other fruit or vegetable in the human diet [8]. However, research still lacks in the area concerning the changes in these constitutions due to the dehydration process and duration involved.

Different drying techniques are available for plums, such as convective drying (CD), vacuum drying, microwave vacuum drying, and freeze drying, which have been used in different studies [1]. Convective drying (CD), where hot air is used as a heat bearer, seems to be a prominent means of moisture removal at a relatively low cost [9] and also gives satisfactory dehydration results with a high drying rate at large capacities [10]. Nonetheless, the degradation of vital nutrient contents, vitamin C, and significant color loss in finished products were observed due to the method's long duration and high-temperature exposure of the fruit samples [11]. Aiming at maintaining the highest product quality, freeze drying (FD) can be contemplated as the leading technique as it retains the greatest portion of vitamins and antioxidant compounds during the dehydration phase [12]. Degradation of beneficial components such as antioxidants and vitamins is practically nonexistent due to the low-temperature drying phase. The drawback of this method is the poor drying rate, which results in a relatively low yield, resulting in significant energy consumption and higher expenses for the use of the refrigeration and vacuum system [13]. Limitations have also been found in vacuum drying and microwave vacuum drying in the production of plum fruit powder [1].

A relatively cheap and simple drying method, the foam mat drying (FMD), has shown advantages over other drying methods such as reduced drying duration and good rehydration properties of the final product [14]. It is also proven to be suitable for drying highly viscous juice or fluid foods with high sugar content [15]. As plum fruit has 9–10% sugar content, foam mat drying would be a viable choice for the dehydration process. In this process, liquid or semiliquid food is whipped with a foaming agent to produce a stable foam by integrating a sufficient proportion of air, resulting in a porous structure with a high rate of heat and mass transfer when dehydrated [16]. The overall process time is greatly reduced in a lower temperature, and thus, a product with better nutritional quality can be obtained [17]. Nonetheless, to have efficiency in this process of drying, the foams must be kept in stable condition both mechanically and thermodynamically in order for the water to be removed effectively while also retaining product quality [18]. So, it is essential to use foaming agents that gives uniform stability. One of the most widely used foaming agents in foam mat drying is egg albumin (EA) which is applied for its protein's ability to form a thick film around air bubbles, resulting in retention of incorporated gases and giving stability on surface tension [19].

In a conventional optimization approach, often known as “one factor at a time” optimization, an individual component is modified constantly while all other remaining factors stay fixed, until the optimal value of response can be chosen. This conventional method is time-consuming and may be incorrect since it does not account for the interplay of variables. This constraint can be easily circumvented by utilizing a certain design of experiment (DOE) [20]. The quality of the finished product depends on a degree of variables; the physical, chemical, and antioxidant properties change and their retention could be the parameter for quality assessment. In the case of heat transfer, the thermal conductivity of the foam is the most affected by an increase in hot air incorporation. Thus, the whipping duration also needs to be considered. As a result, the purpose of this study is to use the response surface technique to assess the influence of the egg albumin content and whipping duration on the physiochemical, antioxidant, and other characteristics of foam mat dried plum fruit powder.

## 2. Materials and Methods

### 2.1. Fresh Plum Fruit Collection

Fresh plum fruits of the *Prunus domestica* variety were collected from Muktagacha, Mymensingh, Bangladesh, which is also known as the common plum. Any visually blemished or visually defected fruits were discarded, and only uniformly ripened fruits were selected for quality purposes. Fresh fruits were then washed with drinkable clean water, cut, removed of stones, processed to make pulp out of them, and stored at 10°C in airtight glass jars until further processing.

### 2.2. Preparation of Plum Foam

To make plum foam, egg albumin was made by beating egg whites and drying them in a cabinet drier at 65°C for around 2 hours or until all moisture was removed. The egg albumin powders were then scraped off, crushed, and stored in airtight glass jars to be mixed with the plum pulp. Plum fruits were juiced using a juicer, and solid portions were discarded to obtain pure plum juice. For each sample preparation, a mass of 100 mL of sample solution was obtained by adding different concentrations of egg albumin. To illustrate, a 2% EA concentration sample was produced by mixing 2 g of EA powder and 98 mL of fresh plum juice together and 4% EA concentration solution was produced by mixing 4 g of EA powder with 96 mL of plum juice. The solution was homogenized and air was incorporated to produce foam using a kitchen mixture (200 W power) at maximum speed. Plum foams of three separate concentrations of 2%, 4%, and 6% of egg albumin were produced with whipping times of 5, 10, and 15 minutes, respectively. The pulp density of the fresh plum juice was measured to be 0.951 g/cm^3^.

### 2.3. Foam Properties

Physical properties of the foam such as foam density, foam expansion, and foam porosity were determined to assess the best foaming conditions.

#### 2.3.1. Foam Density

After foam production, an aliquot of 30 mL was taken in a measuring cylinder at room temperature (25°C). The placement of the foam to the measuring cylinder was done very carefully so that the air incorporated does not evacuate and the foam structure remains intact [21]. The foam density (*ρ*_f_) of each foam were determined using equation (1) of the mass/volume (g/cm^3^) ratio.

(1) $$\begin{array}{c} \rho_{\text{f}}=\frac{\text{weight of foam}\left(\text{g}\right)}{\text{volume of foam}\left(\text{c}{\text{m}}^{3}\right)}. \end{array}$$

#### 2.3.2. Foam Expansion

The expansion of foam is the quantity of incorporated air in the foam during whipping [22]. Foam expansion is determined from the relationship between plum pulp density (*ρ*_p_) and plum foam density (*ρ*_f_) by using equation (2) [23].

(2) $$\begin{array}{c} \text{Foam expansion}\left(\%\right)=\frac{\left(1/\rho_{\text{f}}\right)-\left(1/\rho_{\text{p}}\right)}{1/\rho_{\text{p}}}*100. \end{array}$$

#### 2.3.3. Foam Porosity

The porosity of the foam was also calculated from plum pulp density (*ρ*_p_) and plum foam density (*ρ*_f_) using equation (3) [24]. This equation is only valid when air density is neglected in relating to pulp density (*ρ*_p_). Plum pulp density was determined to be 0.951 g/cm^3^.

(3) $$\begin{array}{c} \emptyset =1-\frac{\rho_{f}}{\rho_{p}}. \end{array}$$

### 2.4. Drying of the Foams

The prepared foams of different concentrations were dehydrated by implementing the foam mat drying technique. After adequate whipping, the foams were evenly spread out on stainless steel trays (35.5^∗^20.5^∗^0.8 cm dimensions) with a thickness of 1.0 cm each and forwarded for drying in a convective oven dryer (Model-Nabertherm TR 1050, Germany) at 65°C temperature with a constant air velocity of 3 m/s, which was determined to be the most efficient temperature and foam thickness for economic foam mat drying with the least loss of vitamins and antioxidants [15, 17, 25, 26]. Higher drying temperatures also decrease moisture and water activity, resulting in a longer shelf life and a lower chance of microbial attack. To determine the drying kinetics with the loss of moisture in plum foam, the samples were weighted with a digital scale every 10 minutes until constant mass was obtained in three subsequent results [27]. Dried foams were then carefully drawn out from the trays and blended to make powder and sieved to pull out particles which were of standard size from 250 to 500 *μ*m to follow-up with further analysis [28]. The powdered samples were vacuum packed in zipper bags and kept at 5°C for further usage.

### 2.5. Analysis of the Dried Plum Powder

#### 2.5.1. Evaluation of Physiochemical Properties

Different physical properties such as the WAI and WSI were studied to the impact of the dehydration conditions of foam mat drying on plum powder. Further, chemical properties of reconstituted plum powder such as total soluble solids, solids, pH, titratable acidity, and the browning index were investigated in order to determine the nutritional value of the fruit powder. 2.5 g of the sample was mixed in 20 mL of water for each chemical analysis.

*(1) Water Solubility Index (WSI)*

For determining the solubility of the powders in water, according to [29], 2.5 g of sample powder was added with 30 mL of distilled water and mixed for 30mins at 30°C. Following that, the solution was centrifuged at 3000 rpm for 30 minutes and the supernatant was carefully placed onto a previously weighted Petri dish and oven dried overnight. Equation (4) was used to compute the percentage of the WSI from the weight of the remaining solids.

(4) $$\begin{array}{c} \text{WSI}\left(\%\right)=\frac{\text{weight of the remaining dry solids}}{\text{weight of the initial powder sample}}*100. \end{array}$$

*(2) Water Absorption Index (WAI)*

The weight of the residual hydrated powder in the centrifuge was also measured using the prior method of determining water solubility for determining the WAI [22]. The water absorption index (WAI) was determined using equation (5).

(5) $$\begin{array}{c} \text{WAI}=\frac{\text{weight of remaining wet solids after centrifuge}}{\text{weight of initial dry powder sample}}. \end{array}$$

*(3) Total Soluble Solids (TSS)*

The TSS was estimated in brix scale using a hand refractometer (Model Atago 2353, MASTER-53M) following the method of Zzaman et al. [30]. Briefly, 1–2 drops of reconstituted sample juice were taken into the glass prism of the refractometer and covered with its plate and reading was taken in face of light.

*(4) pH Determination*

The pH of the samples was measured using a digital pH meter (Model Hanna pH 211, Sigma-Aldrich) following the method adopted by Zzaman et al. [30]. The pH electrode was washed with phosphate buffer solutions (pH = 7.2) after each measurement.

*(5) Titratable Acidity (TA)*

The titratable acidity of the plum juice/reconstituted sample was estimated by adopting the method of Ranganna [31]. A small portion of the powder sample was reconstituted in a beaker with water to a set 10 mL volume. The sample solution was then titrated with 0.1 M NaOH and 2 drops of phenolphthalein as an indicator. Equations (6) and (7) were used to compute the % acidity as anhydrous citric acid.

(6) $$\begin{array}{c} \text{Weight of citric acid}=\frac{0.1\text{ M NaOH}*\text{volume of NaOH }\left(\text{in litre}\right)*192.43}{3}, \end{array}$$

(7) $$\begin{array}{c} \text{Total acidity }\left(\%\right)=\frac{\text{weight of citric acid}}{\text{weight of sample aliquot}}*100. \end{array}$$

*(6) Browning Index (BI)*

The browning index (BI) was calculated spectrophotometrically according to the method of Suh et al. [32]. At pH 1.0, the absorbance of the reconstituted sample was measured at two wavelengths: 510 nm for anthocyanin maximum absorbance and 420 nm for anthocyanin and browning product absorbance. Finally, the BI was computed using equation (8).

(8) $$\begin{array}{c} \text{BI}=\frac{A_{510}}{A_{420}}. \end{array}$$

#### 2.5.2. Antioxidant Properties

*(1) Preparation of Extract*

Four parameters were evaluated to analyze the changes in antioxidant properties of the dried powders in relation to whipping time and egg albumin concentration. Before determining these properties, an extract was prepared from the powders to aid in the analysis. In brief, 1 g of sample powder was dissolved in 25 mL of 80% methanol (80 : 20 *v*/*v*) and thoroughly mixed using a high-speed vortex. The mixture was then centrifuged for 15 minutes at 3000 rpm. The solution was then filtered using Whatman no.1 filter paper, and the filtrate was transferred to a test tube that was stored in the freezer for future examination.

*(2) Determination of the Total Polyphenol Content (TPC)*

The TPC of the sample powders were determined using the Folin–Ciocalteu method following the procedure described by Singleton and Rossi [33] with slight modifications. 1 mL of the extract sample was mixed with an equal quantity of Folin–Ciocalteu reagent and allowed to stand at room temperature for 5 minutes. The solution was then gently mixed with 5 mL of 1 M Na_2_CO_3_, and the total volume of the combination was increased to 10 mL by adding distilled water. After 90 minutes at room temperature, absorbance was measured at 760 nm with a spectrophotometer (Shimadzu UV-1800, Tokyo, Japan). A standard calibrated gallic acid (GA) curve was plotted (*y* = 0.032*x* + 0.090; *R*^2^ = 0.9997) and the measure of TPC was expressed as GA equivalents per gram.

*(3) Determination of the Total Flavonoid Content (TFC)*

TFC of the samples were measured by colorimetric assay [34] with modifications. In brief, 0.25 mL of extract with 0.75 mL of distilled water was taken in a test tube and 5% of 0.15 mL sodium nitrite (NaNO_2_) solution was added to the mixture. Then, 0.3 mL of 10% aluminum chloride (AlCl_3_) solution was added, and, after 5 minutes, 1 mL of 1 M sodium hydroxide solution was added to the mixture. Absorbance of the final mixture was assessed at 510 nm spectrophotometrically (Shimadzu UV-1800, Tokyo, Japan). Again, a calibration curve (*y* = 0.0029*x* + 0.0169) for quercetin was prepared and the TFC was expressed as quercetin equivalent (mg QE/100 g) for the flavonoid contents.

*(4) Determination of Beta Carotene (β-Carotene)*

The beta carotene, like all other carotenoids, is an antioxidant. Beta carotene was determined according to the method of Biswas et al. [35]. 1 g of the powder sample was taken in a test tube and 5 mL of chilled acetone was added with it. After mixing, the sample mixture was vortexed (Model VM2000, Taiwan) using high speed for 15 minutes and centrifuged (Model 416G, Gyrozen, Korea) at 1370 rpm for 10 minutes. Later, the supernatant was separated in another test tube. This process was carried out again with the remaining solids in the previous test tube where 5 mL of chilled acetone was added. After this, the whole supernatant was filtered through Whatman no.1 filter paper. A standard solution was prepared using 0.025 g of standard *β*-carotene (Sigma-Aldrich) mixing with 5 mL of chilled acetone and keeping at a dark place for 10 minutes. The absorbance of the extract and standard solution of *β*-carotene was measured by using a UV-Vis spectrophotometer (Shimadzu UV-1800, Tokyo, Japan) at 449 nm wavelength using equation (9) as follows:

(9) $$\begin{array}{c} \text{Weight of beta carotene in sample }\left(W2\right)=\left(\frac{W1}{X}*Y\right), \end{array}$$

where *W*1 is the weight of the sample, *W*2 is the weight of beta carotene, *X* is the absorbance of the standard beta carotene, and *Y* is the absorbance of the sample.

*(5) Determination of DPPH Radical Scavenging Activity*

The DPPH (2,2-diphenyl-1-picryhydrazal) solution was used to assess the DPPH radical scavenging activity of the aqueous extract according to the procedure as described by Kalantzakis et al. [36]. In brief, a DPPH solution was prepared by adding 0.00394 g DPPH in 100 mL 80% methanol, mixed well and kept in a dark place for about 24 hours. Following that, 1 mL of the extract sample was put in a test tube, accompanied by 4 mL of the freshly produced DPPH solution. The mixture was well mixed and incubated at room temperature for 30 minutes before measuring the absorbance of the incubated solution at 515 nm against a blank (control) with a spectrophotometer (Shimadzu UV-1800, Tokyo, Japan). The radical scavenging activity was measured as a decrease in the absorbance of DPPH and was calculated according equation (10) where *A*_c_ is the absorbance of the control and *A*_s_ is the absorbance of the test sample.

(10) $$\begin{array}{c} \text{DPPH scavenging effect }\left(\%\right)=\frac{A_{\text{c}}-A_{\text{s}}}{A_{\text{c}}}*100. \end{array}$$

### 2.6. Statistical Analysis

Three distinct albumin concentrations and three different whipping times resulted in a total of nine plum powder samples with varying characteristics. An analysis of variance (ANOVA) test was performed on the sample compositions and antioxidant values using SPSS version 20.0 software to find a possible correlation between changes in whipping duration and albumin concentration, and software package R (version 4.1.0) was used for response optimization in this study. All data acquired was gathered through triplicate testing and was reported as mean standard deviation. A difference in *p* values of <0.05 was judged significant.

The experimental design and statistical analysis were carried out using R version 4.1.0 (a statistical computing foundation). Multiple regressions utilizing the least square approach were used to evaluate the data. With three runs at the center, first-order polynomials were used for both the full factorial (2^4^) screening test and the successive evaluation of linear fitness for the full factorial (2^3^) design experiment. The variables (factors) found to be significant (*P* < 0.05) in the screening test were only considered in the subsequent experiments. Equation (11) represents a first-order polynomial equation.

(11) $$\begin{array}{c} y_{r}=a_{o}+\overset{n}{\underset{i=1}{\sum }}a_{i}x_{i}+\overset{n}{\underset{i\neq j=1}{\sum }}a_{ij}x_{i}x_{j}. \end{array}$$

When the first-order models fail to match the experimental data, the Box-Behnken design was used to fit the data with second-order polynomial equations, as shown in equation (12) as follows:

(12) $$\begin{array}{c} y_{r}=a_{o}+\overset{n}{\underset{i=1}{\sum }}a_{i}x_{i}+\overset{n}{\underset{i=1}{\sum }}a_{ii}x_{i}^{2}+\overset{n}{\underset{i\neq j=1}{\sum }}a_{ij}x_{i}x_{j}, \end{array}$$

where *y*_*r*_ denotes the measured response variables and *x*_*i*_ and *x*_*j*_ denote the levels of independent variables. *a*_*o*_ is a constant (predicted reaction in the center), while *a*_*i*_, *a*_*ii*_, and *a*_*ij*_ are the model's linear, quadratic, and two-factor interactive coefficients, respectively. All statistical significance tests were based on the total error criterion, with a confidence level of 95%.

## 3. Results and Discussion

### 3.1. Influence of Egg Albumin Concentration and Whipping Time on the Formation of Plum Foam

The foam properties were measured using three different egg albumin concentrations and three different whipping periods. The quality of foam is important because it influences thermal properties such as air incorporation and flow viability during dehydration, which facilitates economic drying.

#### 3.1.1. Foam Density (FD)

**Figure 1:**
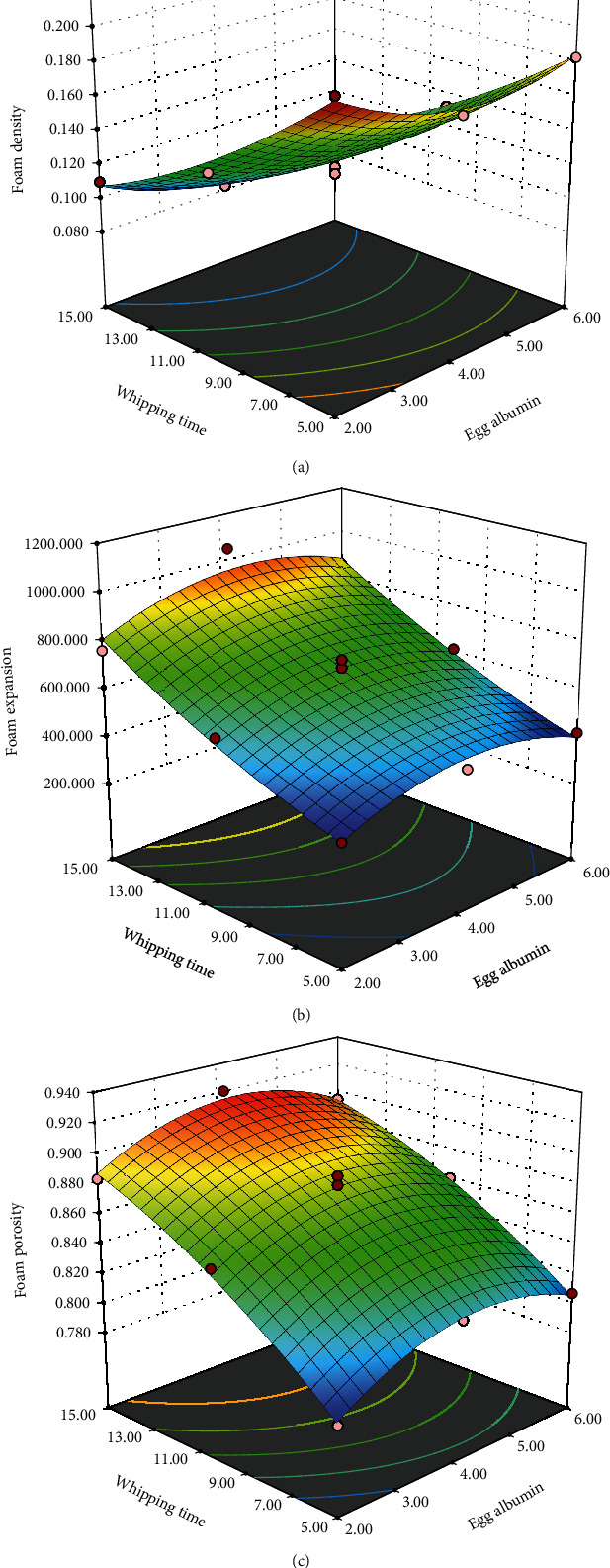
The combined impact of egg albumin and whipping time on foam density (a), foam expansion (b), and foam porosity (c) is depicted by a response surface plot.

The lower the density, the more the foam expands, and this aids in thermal conductivity. As shown in Figure 1, the foam density of the different plum foam products ranged from 0.204 g/cm^3^ to 0.082 g/cm^3^. At the same whipping time, the FD decreased with the increased concentration of egg albumin. A similar decreasing trend in density with an increase in EA concentration was shown in the previous study of cherry foam production [17] and beetroot foam sample [37]. In the case of whipping time, the FD decreased as the whipping duration increased and the lowest FD was observed at a 15-minute duration. A similar decrease in foam density with increased whipping duration was observed in the foam mat drying muskmelon [22]. Lower FD has been shown to facilitate thermal conductivity and, thus, quick and economic drying of the foam. Therefore, a higher concentration of the foaming agent and a longer duration of whipping are recommended. However, too much foaming agent can cause thickening of the foam, which results in increased FD and deformation of the bubbles, as seen in the case of 6% egg albumin concentration. Thus, a moderate level of the foaming agent should be used.

The central composite face center design corresponding responses, foam density (g/cm^3^), foam expansion (%), and foam porosity (g/cm^3^) with *x*_1_ = egg albumin (%) and *x*_2_ = whipping time (min), are shown in Table 1. Regression coefficients, the coefficient of determination (*R*^2^), and lack of fit values for the second-order fitted models corresponding responses, foam density (g/cm^3^), foam expansion (%), and foam porosity (g/cm^3^) with *x*_1_ = egg albumin (%), and *x*_2_ = whipping time (min), are shown in Table 2.

#### 3.1.2. Foam Expansion (FE)

Foam expansion (FE) varied from 366.2% to 1053.3% where the lowest expansion was found in the sample with the lowest whip time and EA concentration, with the highest expansion in foam being 4% egg albumin concentration and 15 minutes of whip time. These values were much higher than those found in previous studies of banana [38], papaya [39], yacon juice [25], and beetroot [37]. So, plum fruit is a good choice for foam mat drying with a foaming agent. Again, a higher concentration of egg albumin (6%) caused a slight decline in the foam expansion, where a concentration of 4% showed the highest expansion in all three durations comparatively. Thus, a moderate concentration of the foaming agent with a higher whipping time is recommended for optimum expansion of foam. The air incorporated through the whipping period gets trapped into the fluid foam as bubbles, which causes the density to decrease as the whipping time increases and consequently causes an increase in foam porosity [38].

The response surface plot showing the combined effect of egg albumin and whipping time on foam density, foam expansion, and foam porosity is shown in Figure 1.

#### 3.1.3. Foam Porosity

Foam porosity is directly interrelated with foam expansion and determines the pore or bubble size in foam. The more air bubbles are trapped in the foam, the greater the foam porosity, and more porosity means more foam expansion. The porosity of the plum foam ranged from 0.7854 to 0.9215. Increased porosity facilitates heated air flow and quick drying of the foam. Again, increased whipping time and a moderate level of egg albumin concentration are advisable to achieve the highest porosity. This porosity rate was directly correlated with foam expansion and density. A previous study on lime juice foam has also shown similar results where expansion, porosity, and stability increased with decreased density of foam [26].

### 3.2. Influence of Egg Albumin Concentration and Whipping Time on the Physiochemical Properties of Plum Powder

The physical and chemical properties of the obtained plum were analyzed to observe the reconstruction capacity of the dried plum powder. These factors are important because the powder must be able to make up for its original juice form again while also retaining its nutritional and sensory characteristics.

#### 3.2.1. Water Solubility Index (WSI)

**Figure 2:**
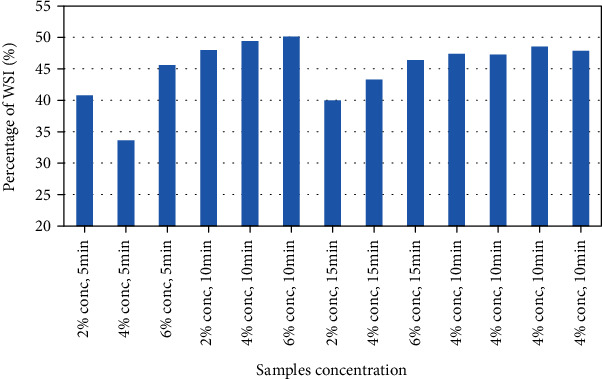
Water solubility index of the samples.

The water solubility of the produced plum powder was found to be 50.1% to 33.6%. The percentage WSI ranged between 40 and 50% for most of the samples shown in Figure 2. WSI is an important factor in indicating the ability of the powder to mix homogenously in water. This factor was not significantly affected by EA concentration or whipping time difference, though there was a slightly increasing trend with increasing EA concentration. These values were similar to the findings of cherry juice powder, which indicated values of 42.4 to 48.4 g/100 g or percentages with 1, 2, and 3% egg white concentration [17]. On the other hand, foam mat dried yacon juice powder showed a much higher (above 80%) solubility index [25], while foam mat dried muskmelon showed a much lower WSI ranging from 23 to 26% [22].

#### 3.2.2. Water Absorption Index (WAI)

**Figure 3:**
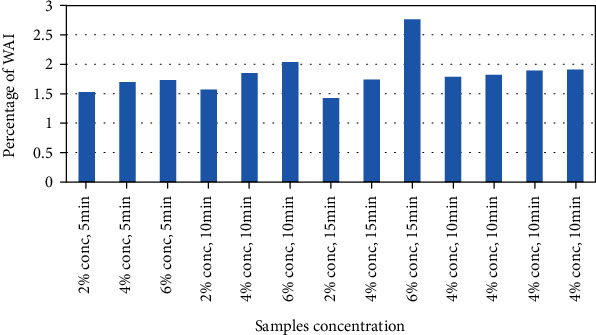
Water absorption index of the sample powders.

The water absorption index (WAI) indicates the capacity of a food substance as to what extent it can absorb water and is directly related to its hydration ability. The WAI ranged between 1.424, the lowest value, and 2.763, the highest value, indicating good rehydration capacity as shown in Figure 3. Increased egg albumin concentration caused a steady increase in the WAI, whereas whipping time does not affect the WAI much, as depicted by the result values shown in Figure 1. A similar trend of an increase in the WAI with increased egg white concentration was also found in the study of lime juice drying [26]. The determined values of this study were in conformity with the values found for yacon juice power, which ranged from 1.181 to 1.81 [25]. On the contrary, Wilson et al. found no significant change in the WAI with an increase in egg white concentration in foam mat drying of mango juice, where the study suggested the opposite that an increase in egg white concentration of 0, 3, 5, 7, and 9% decreased the WAI [40].

#### 3.2.3. Total Soluble Solids (TSS)

**Figure 4:**
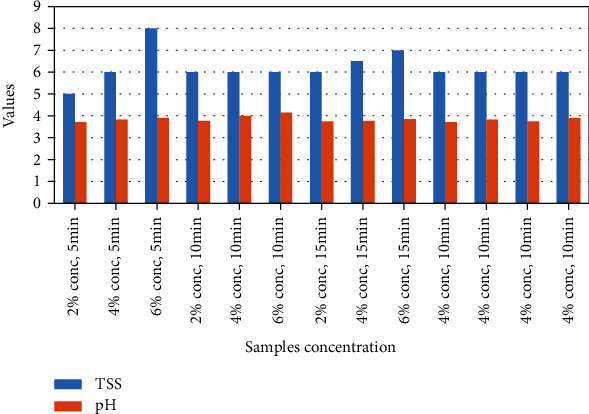
Total soluble solids (TSS) and pH of the sample powders.

The TSS value (°brix) remained almost the same (6°) for most of the samples, ranging from a value of 5° to 8°brix. As observed in Figure 4, increased EA concentration increased the degree of brix. Similarly, TSS for rehydrated yacon juice powder was 8° [25] and 7–7.5° in beetroot powder juice [37]. In the case of beetroot, different concentrations (5 and 10%) of egg white and fish gelatin did not show any significant change in TSS as found in our determinations [37].

#### 3.2.4. pH

Because plum is an acidic fruit, the pH value was bound to be acidic. The values of the reconstituted sample powder ranged from 3.71 to 4.15, as shown in Figure 4. These values were close to the pH value of the fresh plum fruit juice. The increasing EA concentration caused the pH to rise. At each whipping time, there was a significant increase in pH from 2 to 6% concentration. This is due to the higher pH value in the egg white, which is between 7.6 and 8.5 on the pH scale. Abbasi and Azizpour reported a similar trend for cherry juice powder where 1, 2, and 3% egg albumin concentration showed an 11% increase in pH [17]. An increase in whipping duration also caused the pH to rise. Within the same EA concentration, an increase in whipping time increased the pH significantly, especially from 5 minutes to 10 minutes of duration increase. In contrast to that, a whip duration of 15 minutes decreased the pH slightly within the same EA concentration. Overwhipping caused the egg protein denaturation to some degree, which lowered the ability of the egg white to increase the pH of the plum powder.

#### 3.2.5. Titratable Acidity (TA)

**Figure 5:**
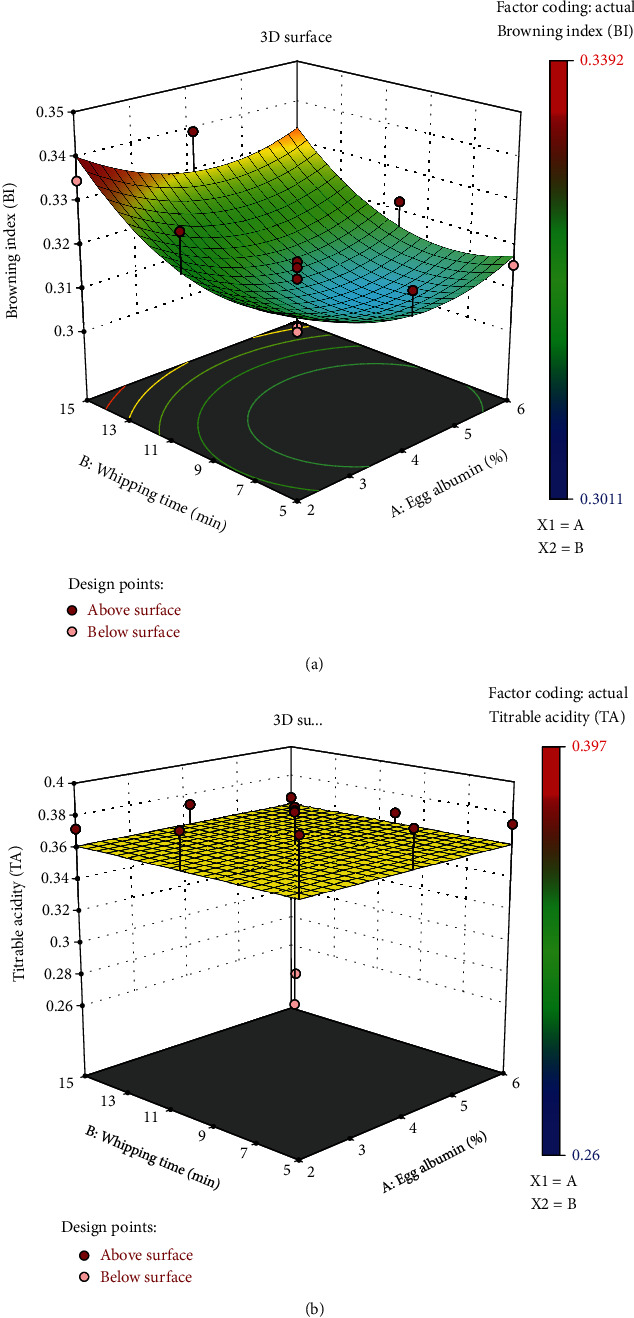
The combined impact of egg albumin and whipping time on BI (a) and TA (b) is depicted by a response surface plot.

The plum powder samples exhibited a considerable amount of titratable acidity owing to the plum fruits' strong acidic composition. The TA of the study samples varied between 0.365 and 0.397. As seen in the result in Figure 5, raising the egg albumin concentration decreased the titratable acidity. This change in acidity with increased EA concentration was statistically significant (*p* < 0.05). Additionally, it was observed that the time of whipping had a diminishing impact on the quantity of TA. Abbasi and Azizpour discovered acidity ranging from 0.224 to 0.329 in foam mats made from dried powder of cherry. It was also reported that an increase in temperature had an increasing effect on total acidity in the same study [17]. A response surface plot showing the combined effect of egg albumin and whipping time on BI (a) and TA (b) is shown in Figure 5.  Table 3 shows the central composite face center design corresponding responses, TA (g%) and BI (%) with *x*1 = egg albumin (%) and *x*2 = whipping time (min), as well as the regression coefficients, the coefficient of determination (*R*^2^), and lack of fit values for the second-order fitted models, corresponding responses, TA (%) and BI (%) with *x*1 = egg albumin (%), presented in Table 4.

#### 3.2.6. Browning Index (BI)

The browning index (BI) is perhaps the most common indicator of nonenzymatic browning or color change in foods with high sugar content where the ratio value of two absorbances is employed as the BI [41]. As the brown color of the examined sample increases, the numerator value at 420 nm increases, which decreases the ratio and ultimately shows a reduced browning index. The BI of these study samples ranged from 0.3124 to 0.3392. From the results obtained, it can be observed that a higher amount of egg albumin concentration decreases the browning effect. According to Abbasi and Azizpour, the egg white is a complete protein and the amino acid structure of the matrix begins the Millard reaction with the reducing sugar, influencing a decrease in the browning index. The browning index showed a decreasing pattern with high egg white concentration in the same study [17], which conforms to our current study of plum fruit. For the whipping time variable, an increase in whipping time with the same EA concentration caused the BI to increase, meaning that less browning occurred. As more whipping disrupts the amino acid structure of egg protein, the Millar reaction is delayed, and thus, less browning occurs. Therefore, increased whipping time is better for the prevention of nonenzymatic browning. However, the BI of plum foam is a factor more affected by drying temperature than any other variable and is more often used as an indicator of quality changes due to thermal treatment [1].

### 3.3. Effect of Egg Albumin Concentration and Whipping Time on the Antioxidant Properties of Foam Mat Dried Plum Powder

#### 3.3.1. Total Polyphenol Content (TPC)

Polyphenols are low-density lipoprotein-type antioxidants and are responsible for the reduction of heart disease. They are a vital antioxidant found in the stoned fruit of prune species, which represents most of the antioxidant content of the fruit [42]. The TPC of the sample extracts ranged from 7.681 mg GAE/g to 12.125 mg GAE/g, which was much greater than the TPC range of regular plum fruits. Michalska et al. reported 0.03 to 4.8 mg/g of TPC for fresh plum juice and a considerably higher TPC of 7.8 to 14.9 mg/g (db) for plum powders obtained using different drying methods, which was similar to the findings of our current study. This was because the plum powder contains the polyphenols in a concentrated amount and, also, mainly due to the fact that the polyphenols found in plum powder belong to the flavanol group, whereas the raw juice polyphenols did not belong to it [7]. From the results in the figure, it was seen that higher egg albumin concentration had an increasing effect on the TPC content, while increased whipping duration lowered the TPC content. However, changes in the TPC with variation in EA concentration and whipping were not too apparent as the incline or decline in TPC was too low. Therefore, whipping time and EA concentration did not affect the amount of TPC to a greater degree. Furthermore, these value changes were not statistically significant.

#### 3.3.2. Total Flavonoid Content (TFC)

Flavonoids are polyphenolic compounds having abilities such as anticancer, anti-inflammatory, and antiviral properties. They make up one-tenth of the total polyphenol content of these stoned prune fruits [43]. The total flavonoid content (TFC) was within the range of 63.827 to 146.59 mg/100 g at the highest. To a certain degree, the TFC was influenced by both variables, EA concentration, and whipping time. At the same time, increased EA concentration caused the TFC to incline, while at the same time, increased whipping time of 15 minutes with greater EA had a lower TFC amount. Sikora et al. reported the flavonoid contents of fresh and frozen *Prunus spinosa* (blackthorn fruit) at 71.75 mg/100 g and 66.78 mg/100 g, respectively. The flavanol content in fresh Claudia plums from Spain was reported to be much higher at 366 mg/100 g of dry weight [44]. The TFC of plum powder reported by Michalska et al. also showed a similar amount, which is congruent with our current study samples.  Table 5 shows the central composite face center design corresponding responses for TPC (GAE/g), TFC (mg/100 g), beta carotene (*μ*g/100 g), and DPPH (%) with *x*_1_ = egg albumin (%) and *x*_2_ = whipping time (min). Regression coefficients, the coefficient of determination (*R*^2^), and lack of fit values for the second-order fitted models corresponding responses, TPC (GAE/g), TFC (mg/100 g), beta carotene (*μ*g/100 g), and DPPH (%) with *x*_1_ = egg albumin (%) and *x*_2_ = whipping time (min), are shown in Table 6.

#### 3.3.3. Beta Carotene (*β*-Carotene)

**Figure 6:**
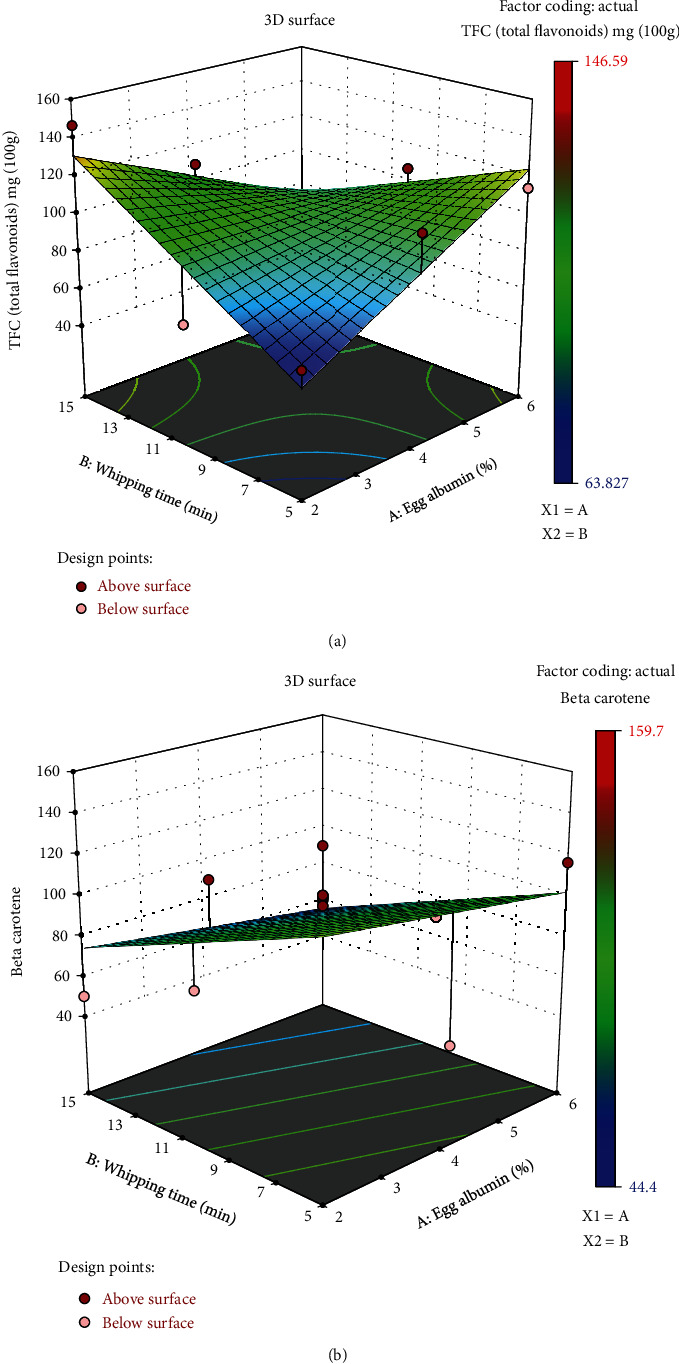
Response surface plot illustrating the combined effect of egg albumin and whipping time on TFC (a) and beta carotene (b).

**Figure 7:**
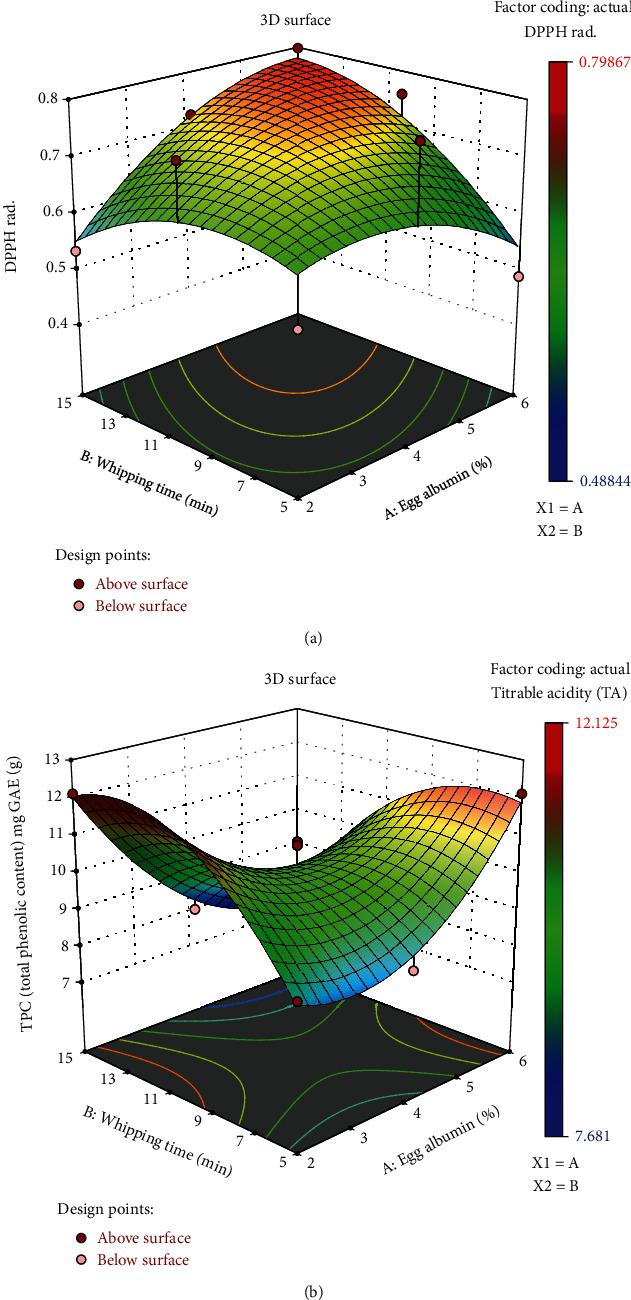
Response surface plot illustrating the combined effect of egg albumin and whipping time on DPPH scavenging activity (a) and TPC (b).

The beta content of the sample powder extracts was in the range of 44.4 to 159.7 *μ*g/100 g. These values were similar to the carotenoid content of fresh plums from California, which ranged from 42 to 109 *μ*g/100 g [45] and *Prunus spinosa* (blackthorn fruit), which was 40 *μ*g/100 g [43]. Thus, there was little to no degradation of carotenes due to the foaming and thermal process of foam mat drying. As seen in Figure 6, the beta carotene concentration was the greatest in the sample with the least amount of EA concentration. Both EA concentration and whipping length seemed to have a negative effect on the beta carotene concentration of the samples. Figures 3 and 4 demonstrate a response surface plot of the combined impact of egg albumin and whipping time on TFC (a), beta carotene TPC (b), DPPH (c), and TPC (d). Figure 6 depicts a response surface plot showing the combined impact of egg albumin and whipping time on TFC (a) and beta carotene (b), whereas Figure 7 depicts a response surface plot showing the combined effect of egg albumin and whipping time on DPPH (c) and TPC (d).

#### 3.3.4. DPPH Radical Scavenging Activity

The DPPH radical scavenging capacity is a popular way of assessing the total antioxidant activity of fruits, and plum fruit generally bears a high quantity of antioxidants. A study reporting the antioxidant capacities of 28 different fruits in Singapore reported plum to be the second highest among them [46]. The percentage DPPH radical scavenging activity of the sample extracts was in the range of 48.844% to 79.867%, which was well within the range of standard plum fruit juice. In a previous study, the antioxidant capacity for the Prunus domestica variety was measured for both fresh and dried samples using methanolic and ethanolic extracts and the highest scavenging activity was reported for the methanolic extract [47]. This study found 87.94 ± 0.81% and 62.40 ± 1.08% DPPH radical scavenging activity for dried and fresh samples of *P. domestica*, respectively, using methanolic extract [47]. Moreover, the previously reported DPPH scavenging activity of fresh plum was 64.62% in Mexican red plum [48].

Therefore, the foam mat drying process did not alter the antioxidant properties of the plum significantly. Again, the high DPPH activity in the dried sample could be due to the increased concentration of the plum. According to Figure 1, the whipping increased the whipping time and caused an increase in DPPH activity. On the other hand, samples with an EA concentration of 4% consistently showed over 70% scavenging activity.

## 4. Conclusions

The influence of whipping duration and the egg albumin content on the quality parameters of foam mat dried plum powder was investigated using response surface modeling, and both factors were shown to have a significant effect on foam characteristics as well as the physicochemical and antioxidant properties of the produced plum powder. In terms of foaming properties, this study indicated that a moderate level of EA concentration (4%) and 10–15 minutes of whipping duration form the best possible foam that facilitates effective drying and thermal conductivity for economic drying. For the antioxidant properties, the foam mat drying process retained them considerably without causing any significant loss. The procedure enhanced the DPPH radical scavenging activity, showing that FMD is one of the best drying techniques. With the addition of more EA, polyphenols, one of the most essential and major part-occupying antioxidants of plum fruit, were increased. The polyphenols were less affected when the whipping time was increased. The effect of the variables on flavonoids was similar to that of the polyphenols, while beta carotene remained unchanged. In the case of DPPH activity, increased whipping time tends to increase the percentage inhibition, and at the same time, 4% EA concentration exhibited the highest free radical scavenging activity. Therefore, the authors conclude that, for an effective and economic dry powder formation using FMD at the industry level, a 4% EA concentration with an increased whipping duration of 10–15 min should be applied to obtain the optimum quality plum powder while retaining the antioxidant properties simultaneously. Because of its nutritious and antioxidative qualities, foam mat dried plum fruit powder may be utilized to make healthy beverage powder because of its nutritious and antioxidative qualities. Chemical stability and microbiological safety can be studied further.

## Figures and Tables

**Table 1:** Central composite face center design corresponding responses, foam density (g/cm^3^), foam expansion (%), and foam porosity (g/cm^3^) with *x*_1_ = egg albumin (%) and *x*_2_ = whipping time (min).

Run no.	*x*_1_ egg albumin	*x*_2_ whipping time	Response foam density	Response foam expansion	Response foam porosity
1	4.00	10.00	924.090	971.740	618.208
2	4.00	15.00	1010.474	872.857	573.617
3	6.00	15.00	388.300	531.303	338.821
4	4.00	5.00	898.352	744.976	485.618
5	4.00	10.00	629.246	789.982	393.355
6	4.00	10.00	787.721	807.360	405.800
7	2.00	15.00	737.156	828.609	541.833
8	4.00	10.00	993.506	967.091	522.293
9	4.00	10.00	925.705	925.803	533.739
10	2.00	5.00	633.823	690.676	349.856
11	6.00	5.00	1065.628	985.797	650.200
12	2.00	10.00	756.961	806.585	531.764
13	6.00	10.00	919.060	924.976	591.927

**Table 2:** Regression coefficients, coefficient of determination (*R*^2^), and lack of fit values for the second-order fitted models corresponding responses, foam density (g/cm^3^), foam expansion (%), and foam porosity (g/cm^3^) with *x*_1_ = egg albumin (%) and *x*_2_ = whipping time (min).

Constants	Predicted foam density	Predicted foam expansion	Predicted foam porosity
Intercept *a*_*o*_	0.0138^∗∗^	3.873*E* + 05^∗∗^	0.0171^∗∗^
Egg albumin *a*_1_	0.0003^∗∗^	5693.87^∗^	0.0003^∗^
Whipping time *a*_2_	0.0119	3.438*E* + 05^∗∗^	0.0139^∗∗^
Egg albumin ^2^*a*_11_	0.0009^∗^	37348.39^∗∗^	0.0016^∗∗^
Whipping time ^2^*a*_22_	0.0002^∗∗^	4202.55^∗^	0.0003^∗∗^
Egg albumin × whipping time *a*_12_	0.0006^∗∗^	371.29^∗∗^	0.0019^∗^
Coefficient of determination (*R*^2^)	0.9749	0.9612	0.9876
*p* value of lack of fit test	0.5857	0.0836	0.5677

^∗^ *p* < 0.05,  ^∗∗^*p* < 0.01, and^∗∗∗^*p* < 0.001.

**Table 3:** Central composite face center design corresponding responses, TA (g %) and BI (%) with *x*_1_ = egg albumin (%) and *x*_2_ = whipping time (min).

Run no.	*x*_1_ egg albumin	*x*_2_ whipping time	Response TA	Response BI
1	4.00	10.00	0.397	0.3194
2	4.00	15.00	0.386	0.3188
3	6.00	15.00	0.374	0.3156
4	4.00	5.00	0.385	0.3315
5	4.00	10.00	0.382	0.3124
6	4.00	10.00	0.369	0.3225
7	2.00	15.00	0.372	0.3348
8	4.00	10.00	0.375	0.3392
9	4.00	10.00	0.365	0.3299
10	2.00	5.00	0.383	0.3151
11	6.00	5.00	0.385	0.3164
12	2.00	10.00	0.26	0.3012
13	6.00	10.00	0.28	0.3011

**Table 4:** Regression coefficients, coefficient of determination (*R*^2^), and lack of fit values for the second-order fitted models corresponding responses, TA (%) and BI (%) with *x*1 = egg albumin (%) and *x*2 = whipping time (min).

Constants	Predicted TA	Predicted BI
Intercept *a*_*o*_	0.1867	0.0011
Egg albumin *a*_1_	0.9627	0.0001
Whipping time *a*_2_	0.0798	0.0004
Egg albumin ^2^*a*_11_	0.9255	0.0001
Whipping time ^2^*a*_22_	0.0002	0.0002
Egg albumin × whipping time *a*_12_	0.1686	3.025*E* − 07
Coefficient of determination (*R*^2^)	0.9315	0.9612
*p* value of lack of fit test	2.04	0.0836

^∗^ *p* < 0.05,  ^∗∗^*p* < 0.01, and^∗∗∗^*p* < 0.001.

**Table 5:** Central composite face center design corresponding responses, TPC (GAE/g), TFC (mg/100 g), beta carotene (*μ*g/100 g), and DPPH (%) with *x*_1_ = egg albumin (%) and *x*_2_ = whipping time (min).

Run no.	*x*_1_ egg albumin	*x*_2_ whipping time	Response TPC	Response TFC	Response beta carotene	Response DPPH
1	4.00	10.00	8.863	65.21	159.7	0.54785
2	4.00	15.00	8.466	110.38	49.2	0.78382
3	6.00	15.00	12.125	114.17	116.7	0.48844
4	4.00	5.00	11.709	63.827	75.6	0.75247
5	4.00	10.00	10.763	94.17	95.6	0.71782
6	4.00	10.00	10.759	106.93	70.3	0.76237
7	2.00	15.00	12.125	146.59	50.2	0.53465
8	4.00	10.00	8.003	109.34	89.8	0.72772
9	4.00	10.00	7.681	72.1	44.4	0.79867
10	2.00	5.00	10.856	87.97	100.8	0.71122
11	6.00	5.00	10.797	92.45	99.6	0.71947
12	2.00	10.00	9.85	95.32	99.5	0.7121
13	6.00	10.00	8.65	94.23	98.4	0.7121

**Table 6:** Regression coefficients, the coefficient of determination (*R*^2^), and lack of fit values for the second-order fitted models corresponding responses, TPC (GAE/g), TFC (mg/100 g), beta carotene (*μ*g/100 g), and DPPH (%) with *x*_1_ = egg albumin (%) and *x*_2_ = whipping time (min).

Constants	Predicted TPC	Predicted TFC	Predicted beta carotene	Predicted DPPH
Intercept *a*_*o*_	25.05	4105.54	3810.71	0.0730
Egg albumin *a*_1_	0.7576	51.47	487.80	0.0077
Whipping time *a*_2_	0.4510	244.10	3322.91	0.0097
Egg albumin ^2^*a*_11_	14.85	6.40	2.37	0.0089
Whipping time ^2^*a*_22_	6.15	0.2407	0.6057	0.0094
Egg albumin × whipping time *a*_12_	6.28	3809.98	4.13	0.0262
Coefficient of determination (*R*^2^)	0.9876	0.9615	0.9562	0.9786
*p* value of lack of fit test	0.6235	0.6355	0.4544	0.2716

^∗^ *p* < 0.05,  ^∗∗^*p* < 0.01, and^∗∗∗^*p* < 0.001.

## Data Availability

Data used and/or analyzed in the study are available from the corresponding author on reasonable request.
